# Genomic epidemiology reveals multiple introductions and spread of SARS-CoV-2 in the Indian state of Karnataka

**DOI:** 10.1371/journal.pone.0243412

**Published:** 2020-12-17

**Authors:** Chitra Pattabiraman, Farhat Habib, Harsha P. K., Risha Rasheed, Pramada Prasad, Vijayalakshmi Reddy, Prameela Dinesh, Tina Damodar, Kiran Hosallimath, Anson K. George, Nakka Vijay Kiran Reddy, Banerjee John, Amrita Pattanaik, Narendra Kumar, Reeta S. Mani, Manjunatha M. Venkataswamy, Shafeeq K. Shahul Hameed, Prakash Kumar B. G., Anita Desai, Ravi Vasanthapuram

**Affiliations:** 1 Department of Neurovirology, National Institute of Mental Health and Neurosciences, Bengaluru, India; 2 TruFactor-InMobi Group, Bengaluru, India; 3 Directorate of Health and Family Welfare Services, Government of Karnataka, Bengaluru, India; Centers for Disease Control and Prevention, UNITED STATES

## Abstract

Karnataka, a state in south India, reported its first case of Severe Acute Respiratory Syndrome Coronavirus 2 (SARS-CoV-2) infection on March 8, 2020, more than a month after the first case was reported in India. We used a combination of contact tracing and genomic epidemiology to trace the spread of SARS-CoV-2 in the state up until May 21, 2020 (1578 cases). We obtained 91 genomes of SARS-CoV-2 which clustered into seven lineages (Pangolin lineages—A, B, B.1, B.1.80, B.1.1, B.4, and B.6). The lineages in Karnataka were known to be circulating in China, Southeast Asia, Iran, Europe and other parts of India and are likely to have been imported into the state both by international and domestic travel. Our sequences grouped into 17 contact clusters and 24 cases with no known contacts. We found 14 of the 17 contact clusters had a single lineage of the virus, consistent with multiple introductions and most (12/17) were contained within a single district, reflecting local spread. In most of the 17 clusters, the index case (12/17) and spreaders (11/17) were symptomatic. Of the 91 sequences, 47 belonged to the B.6 lineage, including eleven of 24 cases with no known contact, indicating ongoing transmission of this lineage in the state. Genomic epidemiology of SARS-CoV-2 in Karnataka suggests multiple introductions of the virus followed by local transmission in parallel with ongoing viral evolution. This is the first study from India combining genomic data with epidemiological information emphasizing the need for an integrated approach to outbreak response.

## Introduction

Severe Acute Respiratory Syndrome Coronavirus 2 (SARS-CoV-2), a novel coronavirus that was first detected in individuals with acute pneumonia in China in late 2019, has now spread throughout the world [1]. The World Health Organization (WHO) on March 11 declared the disease Coronavirus Disease 2019 (COVID-19) caused by SARS-CoV-2 a pandemic [2]. COVID-19 has claimed over 500,000 lives (as of July 5, 2020) and the pandemic is ongoing [3].

Analysis of viral sequences from all over the world is consistent with the emergence of the virus in China in late 2019. This novel virus subsequently spread to Europe, and other parts of the world [4,5]. More than 50,000 complete genomes of SARS-COV-2 are currently available in public databases such as the GISAID initiative (originally known as Global Initiative on Sharing All Influenza Data) [6]. This information is invaluable for understanding evolution of the virus [7], pathogenesis, and design of diagnostic tools. A few studies have combined sequence data with epidemiological data to derive insights on the introduction and spread of the virus in a population.

For instance, a comprehensive study of circulating variants in Iceland highlighted the importation of the virus both from Europe and Southeast Asia [8]. A Study from Guangdong Province in China underscored the importance of importation events into the province and limited local transmission [9]. Whereas a study of viral genomes from the East Coast of the USA combined with travel data revealed the coast-to-coast spread of virus within the country, reiterating the role of domestic travel in the spread of the virus [10]. Initial studies in Washington State uncovered cryptic local transmission emphasizing the need to expand testing and tracing [11]. These studies reiterate the importance of combining sequencing data with public health information.

The first case of COVID-19 in India was detected on January 30, 2020 and case numbers have continued to rise inspite of stringent interventions including nationwide lockdowns. In the first few months of the outbreak, between January 22–April 30, 2020, test results from all over India could be averaged to a positivity rate of 3.9% [12]. Analysis of these cases revealed that the test positivity rate was highest when the samples were from contacts of a known COVID-19 positive case [12].

A large number of SARS-CoV-2 genomes (about 1500 complete genomes as of July 5, source—GISAID) have been sequenced in different parts of India. The first sequences from India were reported from individuals with travel history to China, Italy, and Iran [13,14]. These sequences could be placed in the global context with Nextstrain, an online analysis platform that allows the tracking of pathogen sequences in real time [15].

An analysis of 361 complete genome sequences from India showed that five global clades were circulating in India–old Nextstrain clades B, B4, A2a, A3, and a distinct clade A3i [16]. The A2a (related to GISAID clade G) clade was found to be the most prevalent, followed by A3i [16–18]. While these studies have added valuable information on circulating lineages of SARS-CoV-2 in India, they have not comprehensively linked genomic data with epidemiological information. This study was therefore undertaken to dissect the molecular epidemiology of SARS-CoV-2 in Karnataka, a state in South India. Here we report 91 SARS-CoV-2 genome sequences obtained from individuals who tested positive for the virus by RT-PCR and present an analysis of epidemiological information combined with genomic data to elucidate the introduction and spread of the virus in Karnataka.

## Methods

### Samples and epidemiological data

The Department of Neurovirology, at the National Institute of Mental Health and Neuroscience (NIMHANS), Bengaluru is an ICMR (Indian Council of Medical Research) approved COVID-19 diagnostic centre. Samples received for laboratory diagnosis at this centre between March 5–May 21, 2020, were included in this study. The study was granted a waiver by the Institutional Ethics Committee of NIMHANS in light of the public health emergency. The line list of positive patients was provided by the Directorate of Health and Family Welfare Services, Karnataka and missing data was filled in from the ICMR portal. The line list contained detailed epidemiological workup of each sample including information on age, sex, clinical signs and symptoms, location, contacts, description of exposure type, sampling dates, date of hospitalization and follow up of hospitalized cases. A minimal anonymized data set is provided in S7 Table.

### Shape files and maps

Shape files for the Karnataka District map was provided by Data{Meet} Community Maps Project (http://projects.datameet.org/maps/districts/). It is made available under the Creative Commons Attribution 2.5 India. Shape file for the map of India was obtained from https://github.com/mnitin73/geoIndia and is under MIT License.

### Amplicon sequencing and recovery of SARS-CoV-2 genomes

Samples received for testing at NIMHANS were subjected to RT-PCR based on ICMR guidelines [19]. A total of 21,418 samples were tested in NIMHANS (April 5, 2020–May 20, 2020), 369 of these were positive and 101 were included for sequencing. The criteria for sequencing was RT-PCR positive samples with Ct values under 30. Samples with Ct value >30 were included when they were considered critical for representing a cluster or if they were from symptomatic individuals.

We used a tiling primer based approach for whole genome sequencing described by the ARTIC Network using Primal Scheme [9,20]. Briefly, we used the V3 primer set—these are 96 pairs with amplicons of about 400 basepairs (bp) spanning the whole genome except 31bp of the 5’ and a part of the 3’UTR. PCR was performed by pooling adjacent/overlapping primers into different pools so as to prevent preferential amplification of short fragments between adjacent primer pairs. Primers were initially used at a concentration of 10μM as per the protocol, then modified to amplify regions that were missed by increasing primer concentrations to 50μM. For four regions additional primers were designed (S1 Appendix) and a separate reaction was set up before the pooling step in order to complete the genome. The resulting PCR amplicons were used for preparing libraries for Nanopore sequencing using the native barcoding (NBD 104/114) approach combined with the ligation sequencing kit (SQK-LSK109). Between 12–24 samples were barcoded and included in a single run. The resulting DNA was cleaned up and added to FLO-MIN-106 flow cell and sequenced on the MinION.

### Analysis of sequencing data

Sequences were basecalled and demultiplexed using guppy (v3.6), read lengths between 100–600bp were considered for further analysis. Primers were removed from the sequencing reads by trimming 25bp at the ends and additional trimming based on alignment using BBDuk (v38.37). Resulting reads were mapped to the RefSeq strain (NC_045512) using Minimap2 (v2.17) within Geneious Prime (Geneious Prime 2020.0.3). A consensus was created for regions with coverage >10x using the 0% majority rule and corrected. Consensus was aligned to the reference to ensure the correct reading frame and annotation was transferred from the reference. A schematic of the workflow is provided in S1 Appendix. We obtained 91 genome sequences of these 75 were full-length at 10X (>90% genome coverage) and 16 were partial (>60% genome coverage). We obtained an average of 0.2 million sequencing reads per sample with an idealized coverage of 2810x (S1 Table). SARS-CoV-2 sequences were deposited into the GISAID database and GenBank (NCBI) Sequencing reads have been deposited in the Sequence Read Archive (SRA) under the BioProject PRJNA670824. Accession numbers are provided in S8 Table.

### Phylogenetic analysis, lineage assignment, detection of SNPs and amino acid replacements

Consensus sequences from the 91 genomes from this study were aligned with the reference genome using MUSCLE (v 3.8.425) [21]. The multiple sequence alignment was used to infer the phylogeny using iqtree (v 1.6.12) [22]. A total of 88 DNA models were tested, and the GTR+F+I model was selected based on the Bayesian Information Criterion. Maximum likelihood tree was constructed as the consensus tree from 1000 bootstraps, including the reference sequence (NC_045512) and hCoV19/Wuhan/WH04/2020 as the outgroup. Nodes with bootstrap values >80% were used for interpretation. Time scaled phylogenies were constructed using TreeTime with the multiple sequence alignment described above and the date of sampling as dates. Pangolin lineage assignments were performed using the online tool (Pangolin v2.07 lineages version 2020-08-29) [23]. Lineage assignments from Pangolin were compared with the lineage assignments from the maximum likelihood tree, in case of a discrepancy, sequences from sub-lineages were collapsed to their parent lineage or reassigned based on lineage of sister clades (S6 Table). Single nucleotide polymorphisms (SNPs) and amino acid replacements were detected using the CoV-Glue web application [24]. Both tools use sequences submitted to the GISAID database [6].

### Analysis of epidemiological data and contact map

The epidemiological data was extracted from the line list and a contact map was constructed using the state line list of positive cases. We identified primary and secondary contacts for a patient from the line list. We then built a graph where each node is a positive individual and is connected by an edge with their contacts who were positive. This gives us the contact map. The graphs were then filtered by size of clusters or clusters containing a node with a particular property to focus on clusters of interest. The graphs are visualized using the d3.js [25] library with attributes like clinical state, lineages, and geographical location indicated by colours of the nodes.

## Results

**Fig 1:**
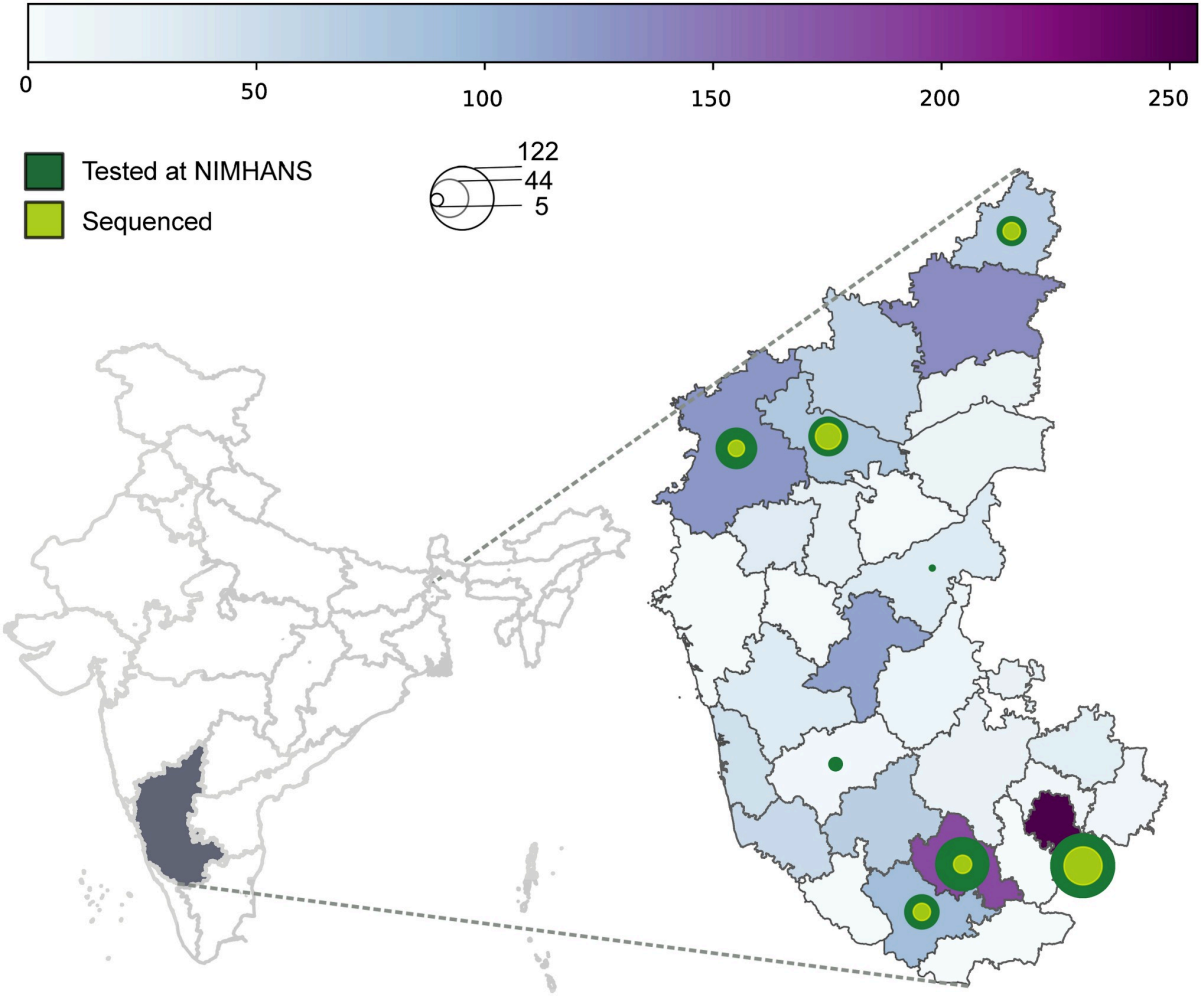
District wise distribution of SARS-CoV-2 positive cases in Karnataka sampled between March 5, 2020 to May 21, 2020. Left—Map of India highlighting the state of Karnataka. Right—Heat map shows the distribution of cases across 30 districts in Karnataka with high burden districts in deep purple. Size of the circle is proportional to the number of cases tested at our centre (green) and the number of cases sequenced (lime). The density of cases are represented by the heat map (horizontal bar) and concentric circles.

Karnataka recorded 1578 cases between March 5–May 21, 2020. Most of these cases were from six high burden districts, with Bangalore Urban (the district encompassing the city of Bengaluru) reporting 256 cases (Fig 1). In total 369 (23.38%) positives were recorded at our centre, of which 101 samples were taken for sequencing (Fig 1, Table 1). The features of positive cases in the Karnataka, and those tested and sequenced at our centre are in Table 1. Most of the positive individuals (1133/1578: 71.8%) in the state were below 40 years of age. More males than females tested positive (987/1578: 62.5%). Amongst the positive individuals in the state, 84.35% were asymptomatic (at sample collection). A total of 87.5% cases (1380/1578) had contact with a known COVID-19 case or travel history. Amongst 369 positive cases tested at our centre, 168 did not have a known contact as of May 21, 2020. We included 24 of these 168 for sequencing.

**Table 1:** Characteristics of positive cases.

		NIMHANS	
	State	Tested	Sequenced
Number of positive cases*	1578	369	101
**Age**			
0–20	374	86	22
20–40	759	197	58
40–60	315	69	15
60–80	126	15	4
**Sex**			
Female (F)	591	120	31
Male (M)	987	249	70
M: F	1.67	2.08	2.26
**Clinical State**			
Asymptomatic** (Asym)	1331	351	91
Symptomatic (Symp)	247	18	10
Ratio Asym**: Symp	5.39	19.50	9.10
**Nature of contact/exposure**			
Contact with COVID-19 case	700	201	66
No known contact			
i. Travel history (international)	96	0	0
ii. Travel history (domestic)	584	104	16
iii. ILI (under investigation)	27	4	2
iv. SARI (under investigation)	55	5	4
v. Under investigation	62	27	9
vi. Contact unknown	54	28	4

Note: Nature of contact/exposure is classified as Contact with COVID-19 case- where tested individual was in contact with a known positive case or No known contact, divided into six categories. i. Travel history (international)—travel history to other countries, ii. Travel history (domestic)—travel within the state or inter-state, iii. ILI (under investigation)—individuals with Influenza like illness with no known source of infection, iv. SARI (under investigation)—individuals with severe acute respiratory infection where the source of infection is not known, v. Under investigation- source of infection is not yet known/contact tracing has not been completed, vi. Contact Unknown- where the tested individual was from a location where there were cases (e.g. a containment Zone) but a specific contact could not be identified.

* Between 5 March 2020–21 May 2020.

** Asymptomatic at sample collection.

Overall 101 samples were sequenced, the Ct values of these samples ranged from 14.5.1–37.85, 91 genomes were obtained. For these 91 samples, the average number of reads was 212322 reads, the average depth of coverage was over 3000x (S1 Table) and 90.48 (average) of the sequenced reads mapped to the reference genome (S1 Table, S1 Fig).

**Fig 2:**
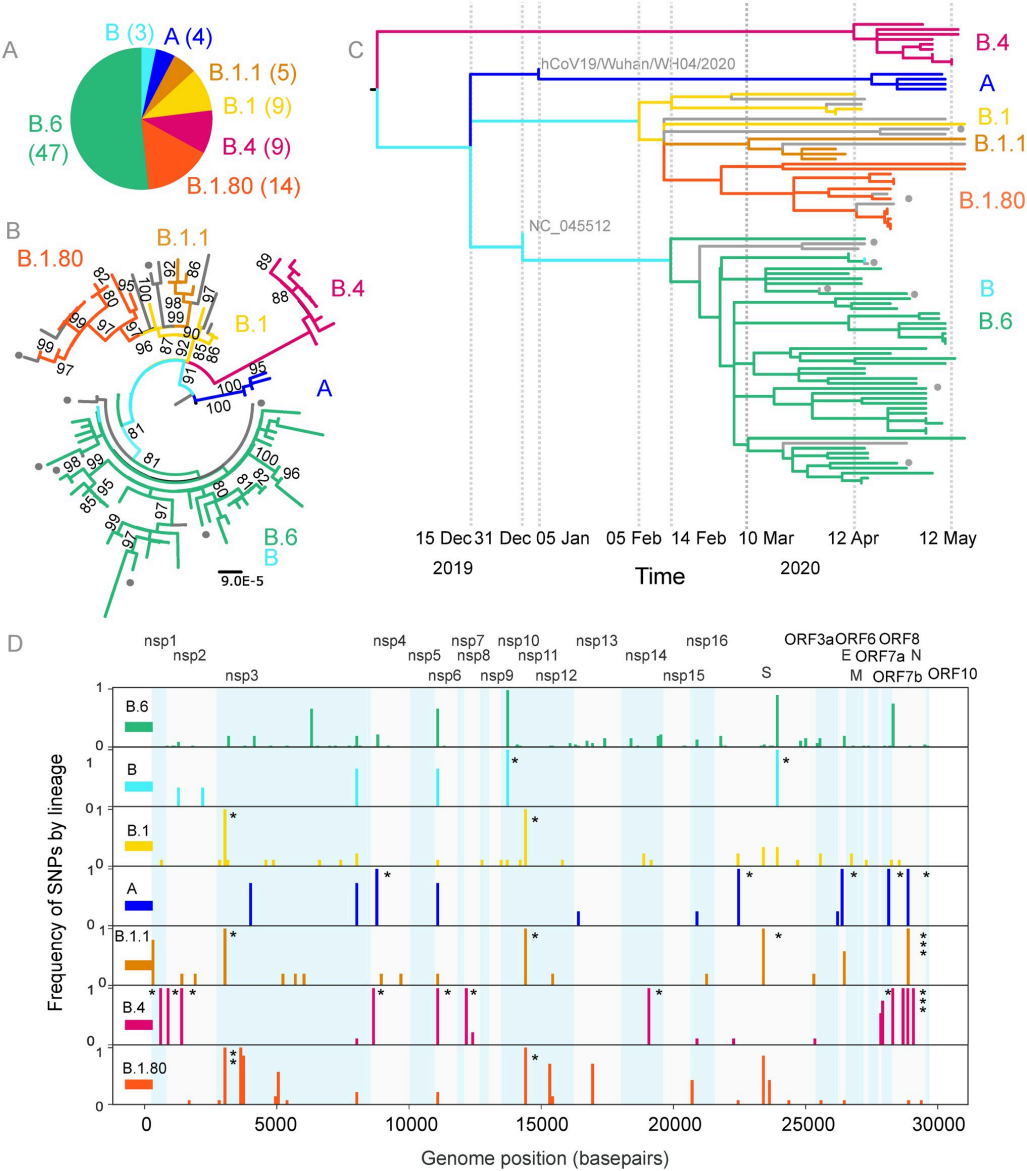
Seven lineages of SARS-CoV-2 circulating in Karnataka. (A) Pie chart shows the proportion of different lineages of 91 SARS-CoV-2 sequences from this study. (B) Maximum likelihood tree was constructed from 91 complete genomes with the reference (NC_045512) and hCoV19/Wuhan/WH04/2020 as the outgroup. Bootstrap support values over 80 are shown. (C) Time scaled maximum likelihood tree of genomes from this study providing a chronology to introduction/importation events and propagation of the lineages post introduction into the state. Sequences are coloured by lineage, grey lines indicate sequences that were reassigned to parent or sister clades. Grey circles indicate sequences from symptomatic individuals. (D) Figure shows SNP frequency for different lineages. The x-axis shows the genome position of the SNP and the y-axes represent the frequency (number of sequences from the lineage that have the SNP/total number of sequences in the lineage). Rows 1–7 represent the seven lineages from Karnataka. Gene boundaries are shaded in blue. Lineage defining SNPs are marked with *. Details of SNPs are provided in S2 Table.

Lineage assignments revealed that the 91 genomes belonged to seven distinct lineages, A(4), B(3), B.1.1(5), B.1(9), B.1.80(14), B.4(9) and B.6(47) (Fig 2A). Six of the seven lineages were apparent by maximum likelihood based phylogeny with bootstrap supports of >80%. The seventh distinct clade has sequences from both lineage B and B.6 clustered together in this analysis (Fig 2B). A time scaled maximum likelihood phylogeny of the genomes with the reference sequence shows that the lineages branched out at different time points (Fig 2C) with defining mutations (S2 Fig, S2 Table).

Overall 154 Single Nucleotide Polymorphisms (SNPs) were identified in the 91 genomes in comparison to the reference sequence (S2 Table). Proportion and position of the SNPs are shown (Fig 2). A total of 100 amino acid replacements were identified (S2 Fig, S3 Table).

**Fig 3:**
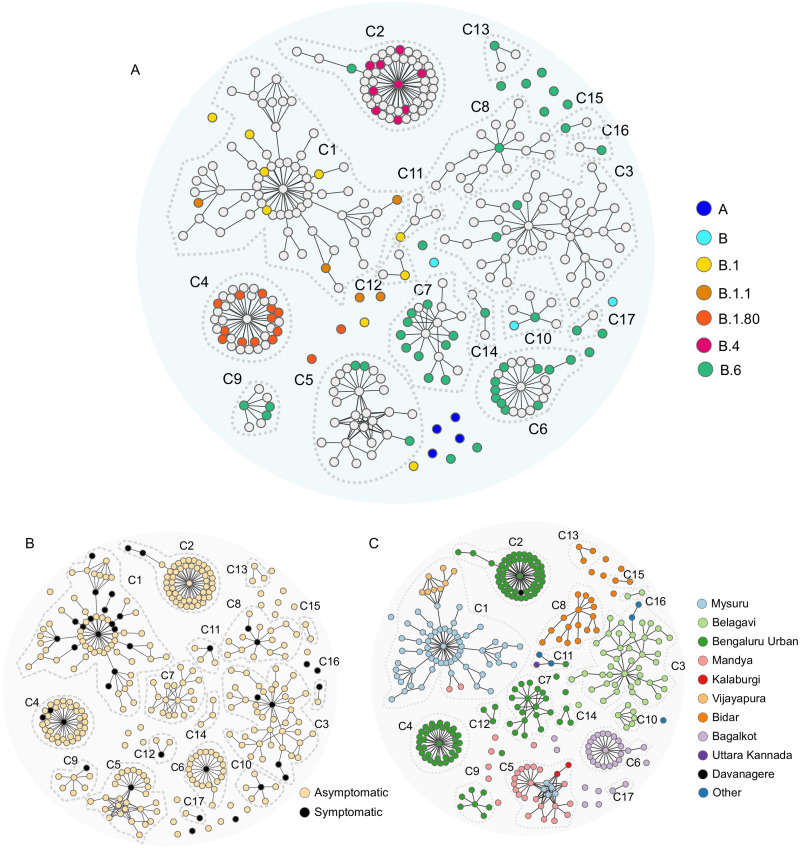
Contact graphs showing lineages, clinical state, and geographical location of clusters. The graphs were made from analysis of contacts from the state line-list of cases and 91 sequences clustered into 17/104 clusters and 24 singletons (individuals with no known contact). These 17 clusters (C1-C17) and 24 singletons (n = 333 individuals) are shown in all the panels (A-C). (A) Contact graph with individuals from whom genomes were obtained are coloured by lineage. Note: Lineages were assigned to all 24 singletons. (B) Contact graph of sequenced clusters and singletons are coloured by clinical status—symptomatic or asymptomatic. Orange depicts symptomatic individuals and black represents the asymptomatic individuals. (C) Graph representing geographic distribution of contact cluster by place of residence. Note: Most of the clusters are restricted to a district. A minority of cases (blue) are from districts other than those listed.

Amongst the 24 sequences from individuals with no known contact (Table 2, Fig 3), four clustered into lineage A and reported travel to other parts of India. Of the remaining 20, six sequences that clustered into lineages B.1.80, B.1, and B.1.1 (two each) also had a history of travel within India. An additional sequence from an individual with no known contact was also assigned the lineage B.1. All five cases (of the 24) which were under investigation belonged to lineage B.6. In addition to this, B.6 was assigned to two individuals with history of travel within India, three sequences from symptomatic individuals and one with contact unknown. In addition, one sequence from a symptomatic individual with SARI and from an individual with no known contact clustered into the B lineage.

**Table 2:** Lineage assignments of positive cases with no known contacts.

Sr. No	Nature of contact/exposure	Lineage	Date of sample collection	District Name/Others
1	Contact unknown	B.1	2020-04-12	Mysuru
2	Contact unknown	B	2020-04-13	Belagavi
3	Contact unknown	B.6	2020-04-14	Others
4	SARI (under investigation)	B	2020-04-15	Bengaluru Urban
5	SARI (under investigation)	B.6	2020-04-16	Bengaluru Urban
6	ILI (under investigation)	B.6	2020-04-25	Bengaluru Urban
7	ILI (under investigation)	B.6	2020-05-04	Bagalkot
8	Under Investigation	B.6	2020-05-08	Bidar
9	Under Investigation	B.6	2020-05-08	Bidar
10	Domestic Travel	A	2020-05-09	Bagalkot
11	Domestic Travel	A	2020-05-10	Bagalkot
12	Domestic Travel	A	2020-05-10	Bagalkot
13	Domestic Travel	A	2020-05-10	Bagalkot
14	Domestic Travel	B.6	2020-05-10	Bagalkot
15	Domestic Travel	B.1	2020-05-10	Bagalkot
16	Under Investigation	B.6	2020-05-10	Bidar
17	Under Investigation	B.6	2020-05-10	Bidar
18	Under Investigation	B.6	2020-05-10	Bidar
19	Domestic Travel	B.1.80	2020-05-16	Mandya
20	Domestic Travel	B.6	2020-05-16	Mandya
21	Domestic Travel	B.1.1	2020-05-16	Mandya
22	Domestic Travel	B.1.1	2020-05-16	Mandya
23	Domestic Travel	B.1	2020-05-16	Mandya
24	Domestic Travel	B.1.80	2020-05-16	Mandya

Analysis of contact information in the state line list, revealed that 822 of 1578 cases could be assigned into 104 contact clusters (S4 Table). Of these 104 clusters, 38 clusters were tested at our centre and 17 of the 38 were included for sequencing. These 17 included 309 people and covered ten large clusters (>5 individuals) from the state (S4 Table).

Of the 17 clusters (C1–C17), C3, C 5–9, C13-17 had sequences which were only assigned to lineage B.6. The cluster C4, had sequences assigned to B.1.80 and clusters C11-12 had sequences assigned to B.1. Three clusters had sequences assigned to more than one lineage. The first cluster, C1 had sequences from B.1. and B.1.1 The second cluster, C2 had a single sequence assigned to B.6 and all other nine sequences belonged to B.4. The cluster C10 had sequences from lineages B and B.6 (Fig 3, S5 Table).

Most of the cases in the state were asymptomatic (1331/1578) (Table 1, Fig 3B). Analysis of information pertaining to the index cases (earliest detected individuals) from the 17 clusters revealed the following—12 of the 17 (70.5%) were symptomatic, of these seven presented with SARI, two with influenza like illness (ILI), three symptomatic individuals had history of inter-state travel (S4 Table, Fig 3) Further, analysis of individuals with maximum number of contacts (spreader) within a cluster revealed that 11/17 were symptomatic (S4 Table, Fig 3).

**Fig 4:**
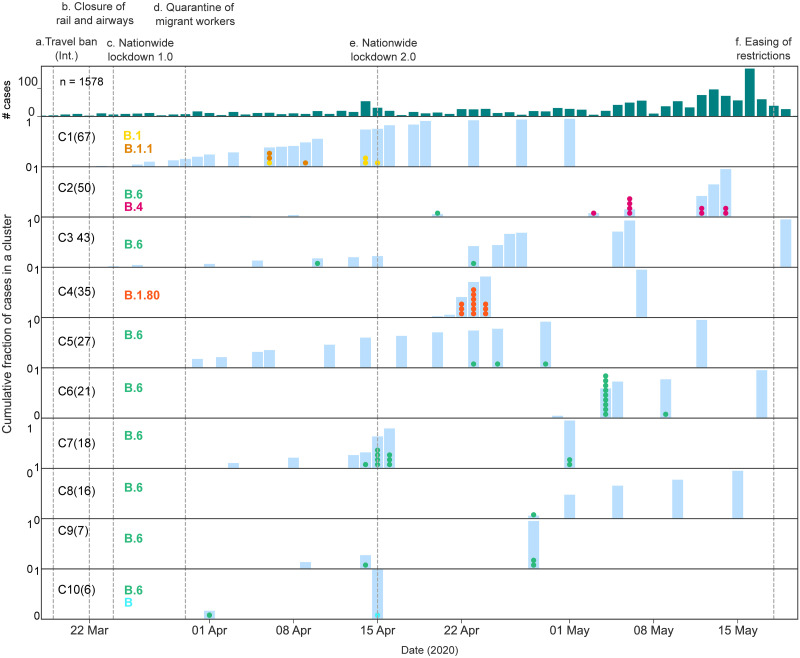
Time course of positive cases in Karnataka by cluster and lineage. The x-axis represents time (March 5-May21, 2020). The y-axis represents number of cases. Note, the first row shows the epi curve, with each bar representing the number of cases recorded on a day while rows 2–11 show the cumulative fraction of cases for the top ten clusters. In rows 2–11, each coloured dot within a vertical bar represents a sequenced sample collected on that date. The dots are coloured by lineage as show in column 2 in the figure. Date of interventions are demarcated by vertical lines and described on top -(a) International travel ban (passenger aircrafts) (b) Closure of domestic travel routes including railways and airways (c) Nationwide lockdown 1.0 (d) Quarantine of migrant workers to restrict movement (e) Nationwide lockdown 2.0 (f) Zone wise easing of movement restrictions.

The location of the clusters was analysed using a contact graph (Fig 3C). Most clusters (12/17) were limited to a single district excepting clusters C1, C2, C3, C5, and C11 which were spread across districts. Time course of the ten largest sequenced clusters showed that cluster C1, C9 and C10 had no cases since May 1, 2020. Clusters C7 and C4 had no linked cases since May 7, 2020. Ongoing transmission was noted for the other clusters (Table 2, Fig 4, S5 Table).

## Discussion

SARS-CoV-2, the virus causing COVID-19, has now spread throughout the world. Despite restricting travel from affected countries early in the pandemic, India started reporting cases of COVID-19 by January 30, 2020 and sustained local transmission was observed in multiple states including Delhi, Maharashtra, and Gujarat [12]. SARS-CoV-2 was first detected in the South Indian state of Karnataka on March 8, 2020 and by May 21, 2020 it had spread to 28 out of the 30 districts of the state resulting in 1578 cases. The data from this study using a combination of genomic epidemiology and contact tracing provides evidence for multiple introductions of the virus into the state with sustained local transmission. We report the circulation of seven lineages of SARS-CoV-2 in the state namely—A, B, B.6, B.1, B.1.80, B.1.1, and B.4 (Pangolin lineage nomenclature). Amongst the 91 virus isolates sequenced in this study, 54.9 (50/91) belong to lineage B and B.6. Most of the contact clusters (14/17) had a single lineage suggestive of multiple introductions of the virus into the state.

Lineage A and B (related to S and L clades of GISAID) of the virus were sequenced in China in January 2020 [23] and they differ at position 8782 in ORF1ab and 28144 in ORF8 respectively. These form the reference sequences and are probably ancestral sequences to other circulating lineages. Viruses from both lineages are now circulating in different countries of the world [23].

In this study, 4 of the 91 sequences, belong to lineage A and were from individuals with travel history to other states within India. This lineage is defined by two SNPs T8782C and C28144T and has been reported from Saudi Arabia, Russia, Turkey, and India [23]. No onward transmission was reported from these four cases, however they indicate continued importation of SARS-CoV-2 into the state emphasizing the need for active surveillance of domestic travel.

Of the lineages in the state, B.1 (related to GISAID clade G, and Nextstrain clade A2a) and B.1.1 (related to GISAID clade GR and Nextstrain clade A2a) are European clades. Both lineages harbour the D614G mutation on the Spike protein. It has been suggested that viruses with this mutation are more infectious and the mutation was present at higher frequency in samples across the world [17,26,27]. Of these two lineages, B.1 was a major contributor to the Italian outbreak [23].

The largest cluster in the state, C1 (comprising of 67 individuals) had four sequenced samples belonging to lineage B.1 and three sequenced samples belonging to lineage B.1.1 (Nextstrain clade A2a). Lineage B.1.1 is defined by three additional (to B.1) SNPs—G28881A, G28882A, G28883C [23]. The cluster C1 was initially restricted to Mysuru and subsequently spread to Vijayapura and Mandya (Fig 3, S5 Table). Of the 67 cases in this cluster, 16 were symptomatic with the index case being a SARI patient (Fig 3). No new cases could be linked to this cluster after May 1, 2020 up until the conclusion of this analysis (May 21, 2020), suggesting that it had been contained.

The index case of this cluster had no history of international travel but was an employee of a company which had a number of international visitors until mid-February 2020, including visitors from Europe. The presence of two lineages is indicative of multiple introductions of the virus in this cluster. Additionally, sequences from five individuals with no known contacts with a positive case were classified into B.1 and B.1.1 (Table 2). Of these four, two each of B.1 and B.1.1 had known history of travel to other states. Highlighting that these lineages are circulating within India and continue to be imported into the state. One other sequence from Mysuru district was also assigned to lineage B.1. However, this sequence clustered separately from all the others in the phylogeny and therefore may represent a separate introduction.

In our study, twelve sequenced samples from a large cluster, C4 (comprising of 35 individuals), belonged to a sub-clade of B.1, B.1.80 which has been sampled from India, Australia, and Luxembourg (Fig 3, S4, S5 and S7 Tables). This cluster was restricted to Bengaluru city and no new cases were reported from it between 7–21 May (Fig 4). The index case for this cluster was a patient with SARI. Two sequences from individuals with recent travel within India also belonged to this lineage. These data suggest that while the initial C4 cluster in the state appears to the be contained, this lineage continues to be imported via domestic travel.

Yet another large cluster (C2) consisting of 50 patients and largely restricted to a quarantine centre in the city of Bengaluru had two lineages B.4 and B.6. Of the sequenced samples from this cluster, nine were assigned to lineage B.4 (Nextstrain A3). B.4 is a clade which was first associated with travellers from Iran and has been sampled from UK, Australia, and India [23]. One sequence from this cluster was from lineage B.6. This cluster merits further analysis as lineage B.4 was unique to this cluster. The presence of two lineages in this cluster have two possible explanations. Either there were dual introductions of the virus (different lineages) into the cluster or that the two lineages are actually part of two clusters. The isolated B.6 case (Figs 3 and 4) and the cluster of B.4 cases (Figs 3 and 4) suggests that the two sets may be epidemiologically unlinked.

Lineages B/B.6 were assigned to the largest number of sequences (50/91) sequences in this study. Using a maximum likelihood based approach we were unable to completely separate B and B.6 as some branches had sequences from both clades (Fig 2). B is the parent lineage for B.6 and it is possible that these three sequences lack the information (S1 Table) for complete classification.

Further, 13 of the 17 clusters in this study and 13 of the 24 cases with no known contact (54.2%) belonged to lineages B/B.6 or both. Lineage B is one of the two clades that were circulating in China in late 2019. Lineage B.6 has earlier been reported from Philippines, UK, North America, Australia, Singapore and has also been reported from other parts of India(16) (Pangolin). The defining mutations for these lineages are similar to that of the A3i clade which has been described as a distinct phylogenetic group in India [16]. Indeed, up to a third of the cases in multiple states across the country belong to the A3i clade [16]. These lineages were detected throughout the study period and across the state, including sequences from two domestic travellers (Fig 4). In Bengaluru city, three clusters C7, C9 and C14 (S5 Table), as well as three symptomatic, epidemiologically unlinked cases were assigned this lineage (Table 2). Overall, our analysis suggests that the B/B.6 lineage is now established and sustained by local transmission in the state with continued importation from other parts of India.

One of the notable features in this study was the ability to assign virus lineages to cases with no known history of contact with a positive individual (Table 2). This underscores the utility of genomic epidemiology in filling the gaps of identifying the source of infection.

Some studies had initially proposed a link between viral lineages, transmission, and disease phenotypes which have not been substantiated by experimental evidence [28]. The analysis of sequences obtained from symptomatic and asymptomatic (at the time of testing) individuals in this study did not reveal any association with a particular lineage. Symptomatic individuals were spread across lineages B.1, B.1.80, and B/B.6 along with asymptomatic individuals (Fig 2, S2 Fig). Of the 17 clusters represented in the sequencing data, both the index case and the spreaders were more often symptomatic (Fig 3, S4 Table). However, sequencing did not reveal any mutations that were specifically associated with clinical state.

Our study was undertaken during the early part of the pandemic during which Karnataka had recorded 1578 positive cases (Fig 4, S7 Table). Within the study period, there was an early ban of international passenger travel. Karnataka has two major international airports (at Bengaluru and Mangaluru) and our data (based on ten of the thirty clusters of >5 individuals in the state) suggests that the tracing and containing of these cases was effective. A nationwide closure of air and rail routes followed. This, however, seems to have been incompletely effective in preventing importation of cases due to domestic travel. In spite of nationwide lockdowns (Fig 4) and quarantining of migrant workers, cases continued to be imported into Karnataka. These observations are consistent with wide-spread circulation of the virus in some states of the country. They emphasize the need for screening and quarantining of travellers as restrictions are relaxed, in addition to the follow up of ongoing transmission in the state (Fig 4).

Our study had the following limitations–it is a single point analysis and some follow-up data is not available, for instance we do not know if individuals who were asymptomatic at testing later developed symptoms. Further, lineage assignments during an outbreak are dynamic and could change as more data is added and sequencing errors are accounted for. Notwithstanding these limitations, our analysis provides insights about introduction, spread, and establishment of SARS-CoV-2 in Karnataka. Further, we were able to capture both geographic diversity and obtain representation from ten large contact clusters in the state. This was made possible by linking epidemiological information to genomic data. Integrating such an approach, in real time, into public health measures is essential for an effective outbreak response.

## Supporting information

S1 Fig Amplicon sequencing of SARS-CoV-2 genomes from PCR positive samples. Relationship between Ct values from RT- PCR and percentage of genome covered at 1X depth (A) and 10x depth (B), total number of reads obtained from a sample (C), percentage of reads that mapped to the reference genome (D) and average depth of sequencing across the genome (E).
(PDF)

S2 Fig Changes in the sequenced genomes. Time scaled maximum likelihood tree of genomes from the study are shown. Colours represent different lineages. Gray lines represent sequences with reassigned lineages and circles represent symptomatic individuals. Key mutations/SNPs for each lineage are shown and detailed in S2 Table. Position of the reference sequence (NC_045512) and an early sequence from Wuhan (hCoV19/Wuhan/WH04/2020) is also shown on the tree (A). Frequency of amino acid replacements across the genome in different lineages are shown. Gene boundaries are shaded in blue. Details of the changes are provided in S3 Table (B). Root to tip regression analysis of sequences in this study is shown. Mutation rate is estimated at 8.764e-04 mutations/site/year with r^2 = 0.24 (C). Note: While the temporal signal in this data is weak, analysis of larger datasets by others is suggestive of a molecular clock.
(PDF)

S1 Table Sequencing results and Ct value of samples. (PDF)

S2 Table Frequency of SNPs in different lineages. (PDF)

S3 Table Frequency of amino acid replacements in different lineages. (PDF)

S4 Table Description of contact clusters. (PDF)

S5 Table Characteristics of sequenced clusters. (PDF)

S6 Table Lineage reclassification. (PDF)

S7 Table Minimal anonymized data set. (CSV)

S8 Table Accession numbers. (PDF)

S1 Appendix Primers and analysis workflow. (PDF)
