# Supplementary material for: Genomic epidemiology reveals multiple introductions and spread of SARS-CoV-2 in the Indian state of Karnataka
Source: PLoS One. 2020 Dec 17;15(12):e0243412. doi: 10.1371/journal.pone.0243412 (PMC7746284; doi:10.1371/journal.pone.0243412)
Supplement: S1 Fig — Relationship between Ct values from RT- PCR and percentage of genome covered at 1X depth (A) and 10x depth (B), total number of reads obtained from a sample (C), percentage of reads that mapped to the reference genome (D) and average depth of sequencing across the genome (E). (PDF) [file pone.0243412.s001.pdf]

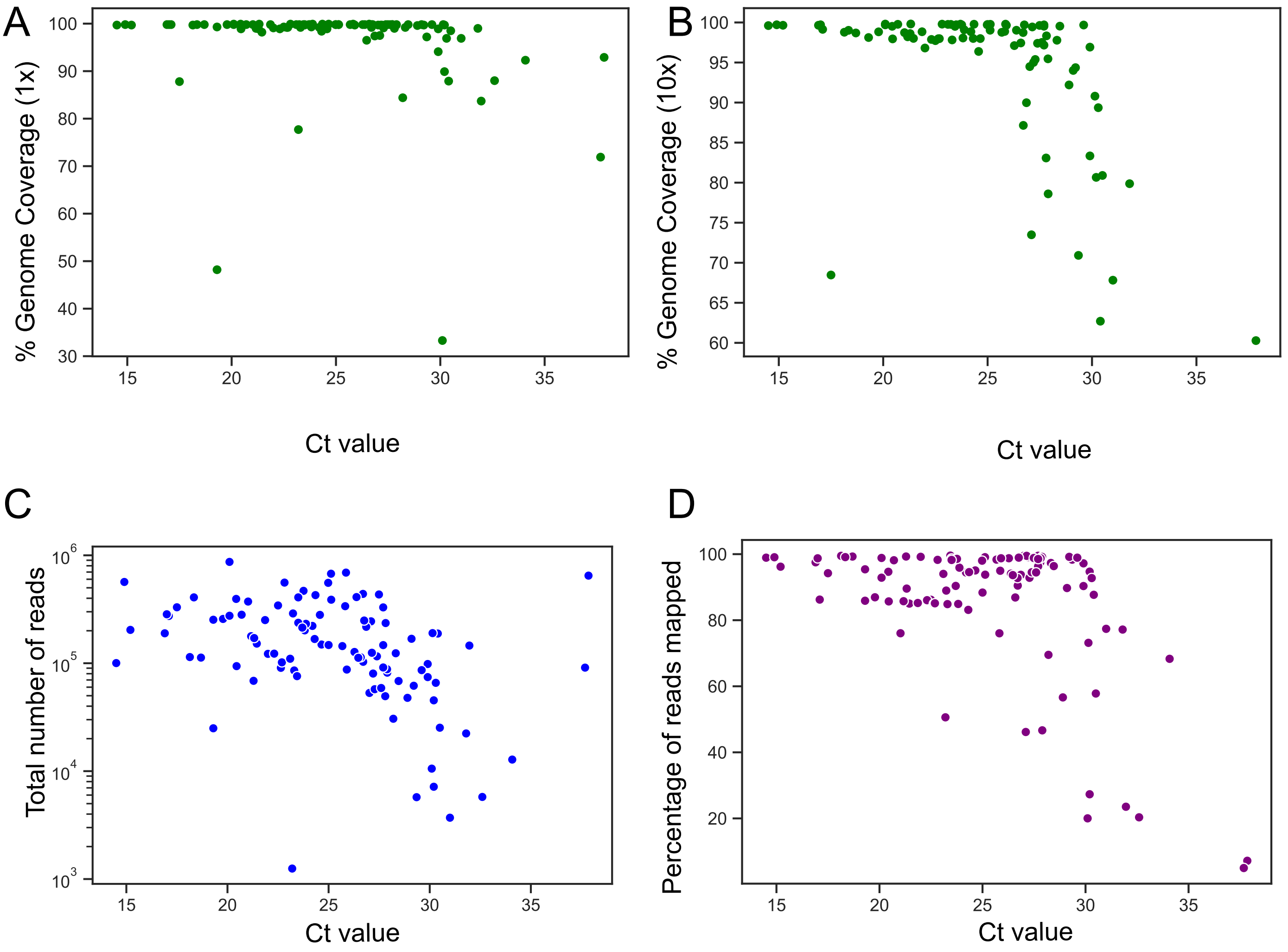

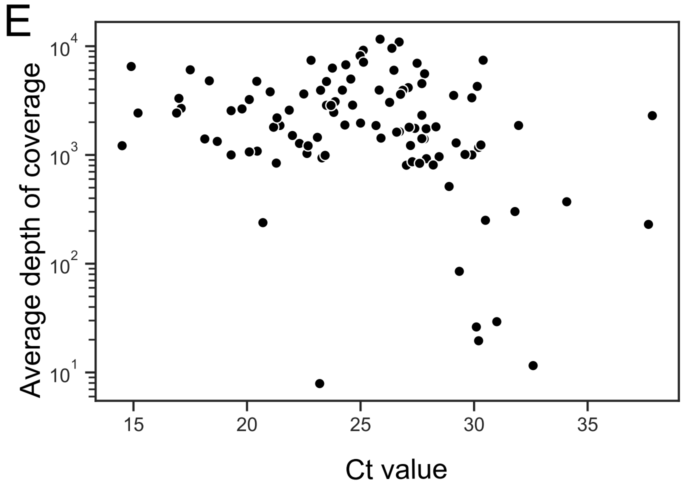

S1 Fig. Amplicon sequencing of SARS-CoV-2 genomes from PCR positive samples. Relationship between Ct values from RT- PCR and percentage of genome covered at 1X depth (A) and 10x depth (B), total number of reads obtained from a sample (C), percentage of reads that mapped to the reference genome (D) and average depth of sequencing across the genome (E).
