# Supplementary material for: Genomic epidemiology reveals multiple introductions and spread of SARS-CoV-2 in the Indian state of Karnataka
Source: PLoS One. 2020 Dec 17;15(12):e0243412. doi: 10.1371/journal.pone.0243412 (PMC7746284; doi:10.1371/journal.pone.0243412)
Supplement: S2 Fig — Time scaled maximum likelihood tree of genomes from the study are shown. Colours represent different lineages. Gray lines represent sequences with reassigned lineages and circles represent symptomatic individuals. Key mutations/SNPs for each lineage are shown and detailed in S2 Table. Position of the reference sequence (NC_045512) and an early sequence from Wuhan (hCoV19/Wuhan/WH04/2020) is also shown on the tree (A). Frequency of amino acid replacements across the genome in different lineages are shown. Gene boundaries are shaded in blue. Details of the changes are provided in S3 Table (B). Root to tip regression analysis of sequences in this study is shown. Mutation rate is estimated at 8.764e-04 mutations/site/year with r^2 = 0.24 (C). Note: While the temporal signal in this data is weak, analysis of larger datasets by others is suggestive of a molecular clock. (PDF) [file pone.0243412.s002.pdf]

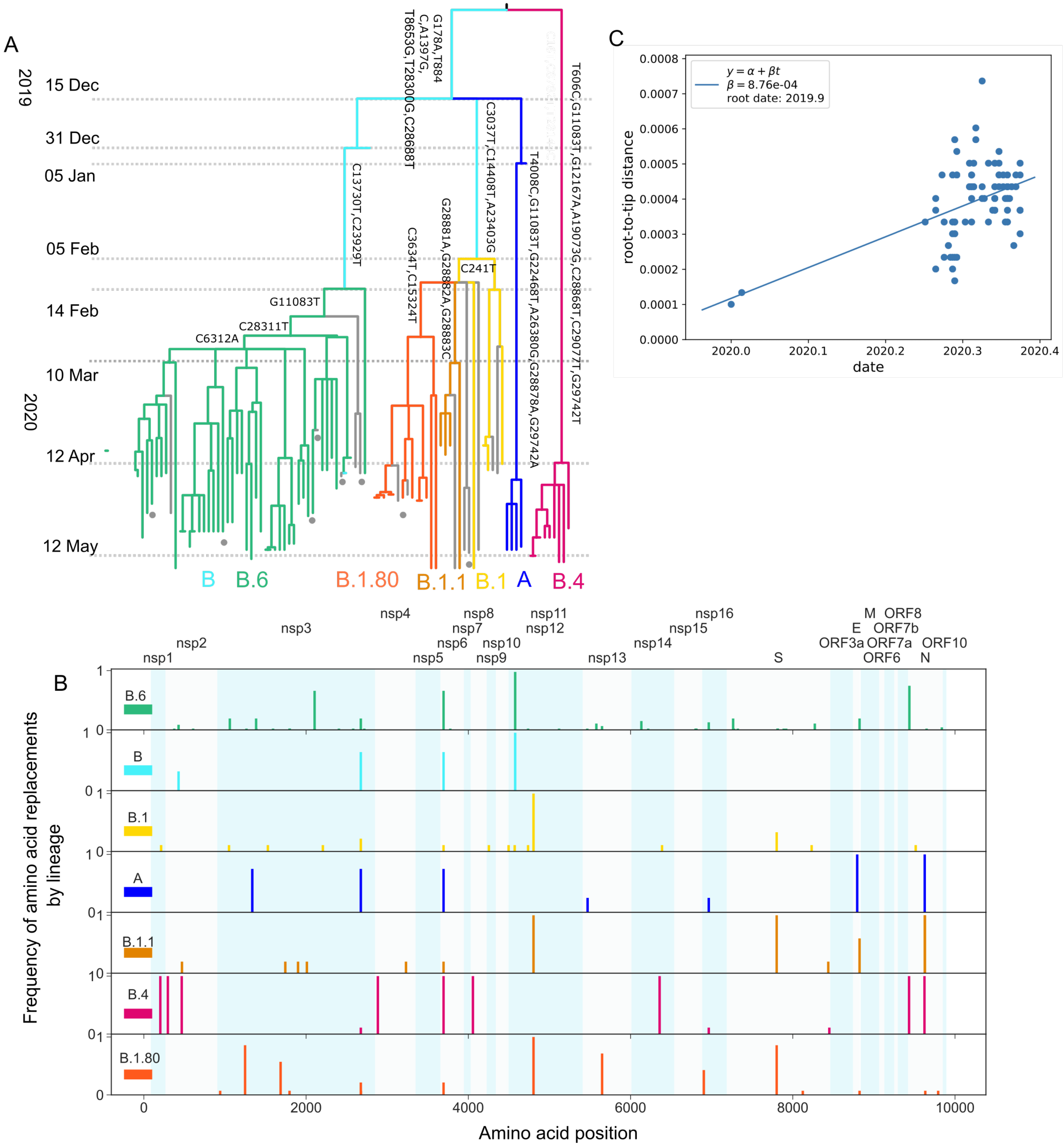

S2 Fig. Changes in the sequenced genomes. Time scaled maximum likelihood tree of genomes from the study are shown. Colours represent different lineages. Gray lines represent sequences with reassigned lineages and circles represent symptomatic individuals. Key mutations/SNPs for each lineage are shown and detailed in S2 Table. Position of the reference sequence (NC_045512) and an early sequence from Wuhan (hCoV19/Wuhan/WH04/2020) is also shown on the tree (A). Frequency of amino acid replacements across the genome in different lineages are shown. Gene boundaries are shaded in blue. Details of the changes are provided in S3 Table (B). Root to tip regression analysis of sequences in this study is shown. Mutation rate is estimated at 8.764e-04 mutations/site/year with $r^2 = 0.24$ (C). Note: While the temporal signal in this data is weak, analysis of larger datasets by others is suggestive of a molecular clock.
