# Supplementary material for: Genomic epidemiology reveals multiple introductions and spread of SARS-CoV-2 in the Indian state of Karnataka
Source: PLoS One. 2020 Dec 17;15(12):e0243412. doi: 10.1371/journal.pone.0243412 (PMC7746284; doi:10.1371/journal.pone.0243412)
Supplement: S1 Table — (PDF) [file pone.0243412.s003.pdf]

S1 Table. Sequencing results and Ct value of samples.

Sr. No	Ct value	# reads	%Mapped	Coverage breadth (%)		Average Depth	Reads
				1X	>10X		
1	37.85	648341	7.17	92.9	60.28	2299.1	648341
2	30.4	188366	87.69	87.9	62.70	7434.6	188366
3	31	3702	77.39	96.9	67.82	29.3	3702
4	17.5	330020	94.23	87.8	68.47	6066	330020
5	29.35	5737	98.33	97.2	70.92	85.06	5737
6	27.1	244455	46.16	97.5	73.49	4161.3	244455
7	27.9	81812	46.66	99.8	78.60	924.3	81812
8	31.8	22354	77.17	99.0	79.87	301.7	22354
9	30.2	45311	94.70	99.7	80.66	1172.4	45311
10	30.5	25255	57.81	98.5	80.90	250.7	25255
11	27.8	49509	97.42	99	83.08	1402.2	49509
12	29.9	98363	90.34	94.1	83.34	3353.2	98363
13	26.71	103092	90.48	99.8	87.15	1634.5	103092
14	27.7	147221	99.50	99.8	99.53	2318.46	26841
15	30.3	65806	92.76	96.9	89.36	1235.0	65806
16	26.86	217498	93.74	97.4	89.98	3930.5	217498
17	30.14	190178	73.15	99.8	90.80	4272.3	190178
18	28.9	47781	56.63	99.6	92.20	513.0	47781
19	29.1	168480	89.74	99.8	94.01	3528	168480
20	29.21	61836	99.18	99.7	94.36	1290.6	61836
21	27.02	53034	99.55	99.7	94.49	804.7	53034
22	27.2	80234	99.11	99.7	95.01	1221.5	80234
23	27.28	57455	92.84	99.1	95.38	866.5	57455
24	27.89	87996	99.17	99.7	95.46	1743.1	87996
25	24.57	280585	95.06	98.9	96.38	4988.3	280585
26	23.24	289260	88.97	99.8	99.76	3934.82	78932
27	22	122157	99.19	99.1	96.81	1509.9	122157
28	29.9	74377	97.21	98.9	96.92	1000.3	74377
29	26.28	127187	98.76	99.8	97.11	3044.4	127187
30	27.7	91365	96.32	99.7	97.17	1413.5	91365
31	21.29	68708	99.28	98.9	98.61	841.46	58884
32	27.39	115854	94.51	99.8	97.40	1756.2	115854
33	26.59	113388	86.88	99.9	97.44	1620.3	113388
34	27.6	58957	94.47	99.8	97.51	837.4	58957
35	22.5	343163	86.18	99.3	97.73	3637	343163

Sr. No	Ct value	# reads	%Mapped	Coverage breadth (%)		Average Depth	Reads
				1X	>10X		
36	28.32	123713	97.33	99.2	97.78	1814.6	123713
37	23.3	85694	84.87	99.2	97.82	939.7	85694
38	22.31	122679	86.06	98.9	97.88	1276.1	122679
39	20.45	93992	85.69	98.9	97.95	1084	93992
40	22.64	90658	84.97	99.2	97.97	1032	90658
41	22.69	101884	85.12	99.2	97.98	1214	101884
42	24.65	149053	95.07	99.9	97.99	2871.7	149053
43	24.31	167748	83.13	98.4	98.00	1884.8	167748
44	21.45	152005	85.03	98.2	98.01	1867	152005
45	23.82	201647	84.92	99.3	98.06	2463	201647
46	19.3	252864	85.89	99.3	98.12	2553.5	252864
47	21.17	177961	85.75	99	98.21	1798.7	177961
48	27.82	235651	98.74	99.6	98.33	5576.4	235651
49	18.69	112746	99.27	99.8	98.69	1332.3	112746
50	21.02	372807	76.05	99.8	98.73	3807.6	372807
51	26.7	439001	92.76	99.2	98.74	10931	439001
52	25.68	144174	98.35	99.8	98.77	1864.8	144174
53	18.14	114088	99.48	99.7	98.78	1403.2	114088
54	19.78	257231	86.94	99.8	98.83	2651	257231
55	21.86	251094	85.21	99.8	98.84	2580.7	251094
56	24.2	221647	94.46	99.2	98.85	3943.2	221647
57	25.82	337778	76.04	99.9	98.88	3950	337778
58	22.82	558746	98.27	99.9	99.79	7422.81	310104
59	18.34	407660	99.10	99.8	99.02	4805.7	407660
60	26.38	410206	94.09	99.8	99.03	9568.5	410206
61	25.12	675238	99.08	99.8	99.03	9176.3	675238
62	23.88	231800	95.90	99.9	99.09	3092.3	231800
63	17.1	274243	86.24	99.8	99.14	2681	274243
64	27.14	125025	99.49	99.7	99.44	1797.6	125025
65	23.44	75911	99.56	99.8	99.51	991.3	75911
66	20.43	394431	94.66	99.9	99.52	4755	394431
67	28.46	68419	96.38	99.8	99.54	964.4	68419
68	14.5	100320	98.93	99.7	99.61	1216.5	100320
69	27.49	433617	98.84	99.8	99.63	6972.5	433617
70	15.2	203680	96.20	99.7	99.65	2429.4	203680
71	16.9	189431	97.53	99.8	99.65	2428.2	189431
72	25.9	87345	98.75	99.7	99.65	1428.3	87345

Sr. No	Ct value	# reads	%Mapped	Coverage breadth (%)		Average Depth	Reads
				1X	>10X		
73	29.6	86291	98.93	99.8	99.68	1010.4	86291
74	26.76	248701	98.94	99.8	99.70	3610	248701
75	23.5	237046	98.66	99.7	99.70	2862.1	237046
76	23.76	468669	98.57	99.8	99.70	6290.4	468669
77	27.7	329180	98.52	99.8	99.70	4531.2	329180
78	20.1	275439	98.85	99.8	99.70	3232.83	275439
79	17	284274	98.74	99.8	99.71	3312.9	284274
80	14.9	566464	99.06	99.8	99.71	6509.5	566464
81	23.5	407632	98.24	99.8	99.71	4737.3	407632
82	24.98	555859	98.02	99.8	99.71	8169	555859
83	25	147496	88.37	99.8	99.75	1963.6	147496
84	23.1	110061	94.02	99.8	99.76	1451.8	110061
85	25.13	387775	93.78	99.8	99.77	7126	387775
86	20.7	281498	98.15	99.8	99.77	238.5	281498
87	24.35	427763	94.56	99.9	99.78	6746	427763
88	23.7	213839	90.38	99.8	99.79	2852.3	213839
89	25.86	691406	94.97	99.8	99.79	11602.8	691406
90	20.1	868971	92.87	99.9	99.80	1066.6	868971
91	21.32	171088	89.57	99.9	99.81	2193	171088
92	34.08	12799	68.31	92.3		371.2	
93	32.6	5766	20.33	88		11.56	
94	30.1	10552	20.02	33.3		26.2	
95	30.2	7166	27.31	89.9		19.56	
96	31.96	145769	23.53	83.7		1866.9	
97	23.2	1251	50.60	77.7		7.9	
98	26.47	112288	93.65	96.5		6009.9	
99	37.68	91043	4.98	71.9		229.9	
100	28.2	30586	69.51	84.4		808.7	
101	19.3	24959	95.41	48.2		1000.6	

Sequencing results of 101 COVID-19 positive samples. Ct Values represent the results of RT-PCR. Total (#) reads is the total number of filtered sequencing reads obtained from each sample. Mapped reads represent the number of reads that aligned to the SARS-CoV-2 reference genome (NC_045512). Percentage (%) of genome covered is shown at the depth of 1X and 10X read depth. Average depth of coverage and lineage assignments (Pangolin ver 2.07) for each sequence are shown. Samples for which genomes were not recovered are shaded in grey.
