# Supplementary material for: Genomic epidemiology reveals multiple introductions and spread of SARS-CoV-2 in the Indian state of Karnataka
Source: PLoS One. 2020 Dec 17;15(12):e0243412. doi: 10.1371/journal.pone.0243412 (PMC7746284; doi:10.1371/journal.pone.0243412)
Supplement: S2 Table — (PDF) [file pone.0243412.s004.pdf]

S2 Table. Frequency of SNPs in different lineages.

Sr. No	SNP	A (4)	B (3)	B.1 (9)	B.1.1 (5)	B.1.80 (14)	B.4 (9)	B.6 (47)
1	C313T				0.80			
2	T606C						1.00	
3	C635T			0.11				
4	C850T							0.02
5	C884T						1.00	
6	G1069A							0.02
7	C1122T							0.02
8	C1281T		0.33					0.09
9	G1397A						1.00	
10	G1408T				0.20			
11	C1684T					0.07		
12	G1820A							0.02
13	C1912T				0.20			
14	C2197T		0.33					
15	G2819T					0.07		
16	C2836T			0.11				
17	C3037T			1.00	1.00	1.00		
18	A3097G			0.11				
19	A3147G			0.11				
20	C3176T							0.19
21	C3634T					1.00		
22	A3742G					0.86		
23	G3791T							0.02
24	T4008C	0.75						
25	G4148T							0.19
26	C4158T							0.02
27	A4584G			0.11				
28	C4776T							0.02
29	A4870G			0.11				
30	T4949C					0.14		
31	C5055T					0.57		
32	G5230T				0.20			
33	C5385G					0.07		0.02
34	A5390G							0.02
35	C5392G							0.02

Sr. No	SNP	A (4)	B (3)	B.1 (9)	B.1.1 (5)	B.1.80 (14)	B.4 (9)	B.6 (47)
36	T5394C							0.02
37	C5700A				0.20			
38	C6027T				0.20			
39	C6310A							0.02
40	C6312A							0.66
41	C6541T							0.02
42	G6617T			0.11				
43	C6997A							0.02
44	T7211C							0.02
45	C7420T			0.11				
46	G7739A							0.02
47	G7975T							0.02
48	T8022G	0.75	0.67	0.22		0.21	0.11	0.19
49	C8025T					0.07		
50	A8026T	0.25				0.07		0.02
51	T8151C							0.02
52	G8653T						1.00	
53	C8782T	1.00						
54	C8818T							0.21
55	C8950T				0.20			
56	G9211A							0.02
57	C9693T				0.20			
58	G11083T	0.75	0.67	0.11	0.20	0.21	1.00	0.66
59	G11330A							0.02
60	G12167A						1.00	
61	C12406T						0.22	
62	T12760A			0.11				
63	C13487T			0.11				
64	A13712G			0.11				
65	C13730T		1.00					0.98
66	C14076T							0.02
67	C14097T							0.04
68	A14207C,A14207T			0.11				0.02
69	C14408T			1.00	1.00	1.00		
70	C15324T					0.71		
71	C15352T							0.02

Sr. No	SNP	A (4)	B (3)	B.1 (9)	B.1.1 (5)	B.1.80 (14)	B.4 (9)	B.6 (47)
72	A15435G				0.20	0.14		0.02
73	T15804C			0.11				
74	C16092T							0.06
75	C16293T							0.04
76	G16396A							0.02
77	A16408G	0.25						
78	T16732G							0.11
79	G16943T							0.06
80	G16945A					0.71		
81	C17403T							0.15
82	G18397T							0.15
83	G18651T							0.02
84	C18877T			0.22				
85	A19073G						1.00	
86	G19162T			0.11				
87	A19422G							0.19
88	C19524T							0.21
89	C20402T							0.02
90	G20419T							0.02
91	A20435G							0.02
92	G20709T					0.43		
93	T20891G	0.25					0.11	0.13
94	A20895T							0.02
95	C20896G							0.02
96	G21255T				0.20			
97	A21792T							0.19
98	G21824C							0.02
99	T21965C							0.02
100	T22276C						0.11	
101	C22444T			0.22				
102	C22450T					0.07		
103	G22468T	1.00						
104	C23320T							0.02
105	A23403G			0.33	1.00	0.86		
106	T23431A							0.02
107	T23433C							0.02

Sr. No	SNP	A (4)	B (3)	B.1 (9)	B.1.1 (5)	B.1.80 (14)	B.4 (9)	B.6 (47)
108	G23441C							0.04
109	A23442C							0.02
110	C23635T					0.43		
111	G23663T							0.02
112	G23755T							0.02
113	C23929T		1.00	0.33				0.89
114	G24368C					0.07		
115	G24697T			0.11				
116	G24812T							0.11
117	C25006T							0.15
118	G25311T				0.20			
119	C25350T						0.11	
120	G25437T							0.06
121	C25546A							0.15
122	G25563T			0.22				
123	C25584T					0.07		
124	G26211T	0.25						
125	A26380G	1.00						
126	G26467T				0.60	0.07		0.19
127	C26735T			0.22				
128	C26801T							0.02
129	G27204T							0.02
130	C27297T			0.11				
131	G27378T							0.02
132	C27847T						0.56	
133	G27915T						0.78	
134	G27990T							0.02
135	T28144C	1.00						
136	G28188C							0.02
137	A28247C			0.11				
138	C28253T			0.11				
139	G28300T						1.00	
140	C28311T							0.74
141	C28545T			0.11				
142	T28688C						1.00	
143	C28868T						1.00	

Sr. No	SNP	A (4)	B (3)	B.1 (9)	B.1.1 (5)	B.1.80 (14)	B.4 (9)	B.6 (47)
144	G28878A	1.00						
145	G28881A				1.00			
146	G28882A				1.00			
147	G28883C				1.00			
148	G28899T					0.07		
149	G28946T							0.02
150	C29077T						1.00	
151	C29376T					0.07		
152	G29511T							0.04
153	G29540A							0.02
154	T29623A							0.02

Single nucleotide polymorphisms (SNPs) were identified in the genomes with respect to the reference sequence (NC_045512). The frequency of SNPs was calculated by the number of sequences with the SNP/total number of sequences in the lineage. SNPs are ordered by their position on the genome. Number of sequences in each lineage are indicated in brackets in the header.
