# Supplementary material for: Genomic epidemiology reveals multiple introductions and spread of SARS-CoV-2 in the Indian state of Karnataka
Source: PLoS One. 2020 Dec 17;15(12):e0243412. doi: 10.1371/journal.pone.0243412 (PMC7746284; doi:10.1371/journal.pone.0243412)
Supplement: S3 Table — (PDF) [file pone.0243412.s005.pdf]

S3 Table. Frequency of amino acid replacements in different lineages.

Sr. No	Amino Acid Replacement (Gene-change)	A (4)	B (3)	B.1 (9)	B.1.1 (5)	B.1.80 (14)	B.4 (9)	B.6 (47)
1	E-I46V	1.00						
2	E-V75F				0.60	0.07		0.19
3	N-D225Y							0.02
4	N-G204R				1.00			
5	N-P13L							0.74
6	N-P199S						1.00	
7	N-P368L					0.07		
8	N-Q9H						1.00	
9	N-R203K				1.00			
10	N-R209I					0.07		
11	N-S202N	1.00						
12	N-S413I							0.04
13	N-T91I			0.11				
14	ORF 10-N22K							0.02
15	ORF 3a-L15F							0.06
16	ORF 3a-L52I							0.15
17	ORF 3a-Q57H			0.22				
18	ORF 6-E59D							0.02
19	ORF 6-M1I							0.02
20	ORF 7b-S31L						0.56	
21	ORF 8-G8*						0.78	
22	ORF 8-L118F			0.11				
23	ORF 8-L84S	1.00						
24	ORF 8-V33F							0.02
25	ORF 8-V99L							0.02
26	S-A701S							0.02
27	S-C1250F				0.20			
28	S-D1084Y							0.11
29	S-D614G			0.33	1.00	0.86		
30	S-D627H							0.02
31	S-D627P							0.02
32	S-D88H							0.02
33	S-D936H					0.07		
34	S-F135L							0.02

Sr. No	Amino Acid Replacement (Gene-change)	A (4)	B (3)	B.1 (9)	B.1.1 (5)	B.1.80 (14)	B.4 (9)	B.6 (47)
35	S-I624T							0.02
36	S-K1045N			0.11				
37	S-K77M							0.19
38	S-M731I							0.02
39	S-P1263L						0.11	
40	nsp1-I114T						1.00	
41	nsp1-R124C			0.11				
42	nsp12-A16V			0.11				
43	nsp12-A97V		1.00					0.98
44	nsp12-H256L			0.11				
45	nsp12-H256P							0.02
46	nsp12-K91R			0.11				
47	nsp12-L638F							0.02
48	nsp12-P323L			1.00	1.00	1.00		
49	nsp13-A237T					0.71		
50	nsp13-G54S							0.02
51	nsp13-S166A							0.11
52	nsp13-S236I							0.06
53	nsp13-T58A	0.25						
54	nsp14-D345G						1.00	
55	nsp14-D375Y			0.11				
56	nsp14-E204D							0.02
57	nsp14-V120L							0.15
58	nsp15-D267Y							0.02
59	nsp15-D272G							0.02
60	nsp15-S261L							0.02
61	nsp16-M17I					0.43		
62	nsp16-P80A							0.02
63	nsp16-V78G	0.25					0.11	0.13
64	nsp2-A159V		0.33					0.09
65	nsp2-E201D				0.20			
66	nsp2-G339S							0.02
67	nsp2-P106L							0.02
68	nsp2-R27C						1.00	
69	nsp2-V198I						1.00	
70	nsp3-A1769V					0.07		

Sr. No	Amino Acid Replacement (Gene-change)	A (4)	B (3)	B.1 (9)	B.1.1 (5)	B.1.80 (14)	B.4 (9)	B.6 (47)
71	nsp3-A358S							0.02
72	nsp3-A480V							0.02
73	nsp3-A889G					0.07		0.02
74	nsp3-A994D				0.20			
75	nsp3-D1674N							0.02
76	nsp3-D622G			0.11				
77	nsp3-E143G			0.11				
78	nsp3-F430S	0.75						
79	nsp3-F892S							0.02
80	nsp3-G1300C			0.11				
81	nsp3-I341M					0.86		
82	nsp3-K837N				0.20			
83	nsp3-N891E							0.02
84	nsp3-P1103L				0.20			
85	nsp3-P153S							0.19
86	nsp3-S1197R							0.02
87	nsp3-T1198K							0.66
88	nsp3-T686I							0.02
89	nsp3-T779I					0.57		
90	nsp3-V1768G	0.75	0.67	0.22		0.21	0.11	0.19
91	nsp3-V1811A							0.02
92	nsp3-V34L					0.07		
93	nsp3-V477F							0.19
94	nsp3-W1498R							0.02
95	nsp4-A380V				0.20			
96	nsp4-M33I						1.00	
97	nsp6-L37F	0.75	0.67	0.11	0.20	0.21	1.00	0.66
98	nsp6-V120I							0.02
99	nsp8-V26I						1.00	
100	nsp9-D25E			0.11				

Number of sequences in each lineage are indicated in brackets.
