# Supplementary material for: Genomic epidemiology reveals multiple introductions and spread of SARS-CoV-2 in the Indian state of Karnataka
Source: PLoS One. 2020 Dec 17;15(12):e0243412. doi: 10.1371/journal.pone.0243412 (PMC7746284; doi:10.1371/journal.pone.0243412)
Supplement: S4 Table — (PDF) [file pone.0243412.s006.pdf]

S4 Table. Description of Contact Clusters.

		Our Centre	
	**State**	**Tested**	**Sequenced**
Total No of contact clusters	104	38	17
Total No. of people connected	822	398	309
Size of cluster			
0 – 5	74	22	7
> 5	30	16	10
**Nature of contact/exposure of index case***			
Contact with COVID-19 case	0	0	0
No known contact			
i) Travel History (International)	14	0	0
ii) Travel History (Domestic)	20	10	5
iii) SARI (under investigation)	29	9	7
iv) ILI (under investigation)	19	4	2
v) Under Investigation	10	8	3
vi) Contact unknown	12	7	0
**Clinical state of index case**			
Asymptomatic*(Asym)	29	19	5
Symptomatic (Sym)	75	19	12
Ratio (Asym: Sym)	0.39	1.00	0.42
**Nature of contact/exposure of spreader****			
Contact with COVID-19 case	12	4	3
No known contact			
i) Travel History (International)	14	0	0
ii) Travel History (Domestic)	16	9	4
iii) ILI (under investigation)	19	4	2
iv) SARI (under investigation)	22	7	5
v) Under Investigation	10	8	3
vi) Contact unknown	11	6	0
**Clinical state of spreader**			
Asymptomatic (Asym)	35	20	6
Symptomatic (Sym)	69	18	11
Ratio (Asym: Sym)	0.51	1.11	0.55

* earliest detected individual from a cluster, ** individual with maximum number of connections in a cluster,

Note: Nature of contact/exposure is classified as Contact with COVID-19 case- where tested individual was in contact with a known positive case or No known contact, divided into six categories. i. Travel history (international) - travel history to other countries, ii. Travel history (domestic) - travel within the state or interstate, iii. ILI (under investigation) - individuals with Influenza like illness with no known source of infection, iv. SARI (under investigation) - individuals with severe acute respiratory infection where the source of infection is not known, v. Under investigation- source of infection is not yet known/ contact tracing has not been completed, vi. Contact Unknown- where the tested individual was from a location where there were cases (eg. containment zone) but a specific contact could not be identified.
