# Supplementary material for: Genomic epidemiology reveals multiple introductions and spread of SARS-CoV-2 in the Indian state of Karnataka
Source: PLoS One. 2020 Dec 17;15(12):e0243412. doi: 10.1371/journal.pone.0243412 (PMC7746284; doi:10.1371/journal.pone.0243412)
Supplement: S5 Table — (PDF) [file pone.0243412.s007.pdf]

S5 Table. Characteristics of sequenced clusters.

Cluster	No. of people	Lineage *	Age Distribution					Clinical State**		District
			0-20	20-40	40-60	60-80	80-100	Asym	Sym	
C1	67	B.1(4), B.1.1(3)	7	46	11	3	0	51	16	Mysuru (57), Vijayapura (8), Mandya (2)
C2	50	B.4 (9), B.6(1)	10	37	2	1	0	48	2	Bengaluru Urban (49), Davanagere (1)
C3	43	B.6 (2)	10	18	13	2	0	39	4	Belagavi (41), Tumkur (2)
C4	35	B.1.80 (12)	4	23	7	0	1	32	3	Bengaluru Urban (35)
C5	27	B.6 (3)	7	14	5	1	0	26	1	Mandya (20), Mysuru (5), Kalaburgi (2)
C6	21	B.6 (10)	4	10	5	2	0	20	1	Bagalkot (21)
C7	18	B.6 (10)	1	9	8	0	0	18	0	Bengaluru Urban (18)
C8	16	B.6 (1)	3	6	5	1	1	14	2	Bidar (16)
C9	7	B.6 (3)	4	0	2	1	0	6	1	Bengaluru Urban (7)
C10	6	B (1), B.6 (1)	1	4	1	0	0	5	1	Belagavi (6)
C11	4	B.1 (1)	1	1	2	0	0	3	1	Bengaluru Urban (1), Uttara Kannada (1), Others (2)
C12	3	B.1 (1)	1	2	0	0	0	2	1	Bengaluru Urban (3)
C13	3	B.6 (1)	2	1	0	0	0	3	0	Bidar (3)
C14	3	B.6 (1)	2	1	0	0	0	3	0	Bengaluru Urban (3)
C15	2	B.6 (1)	1	1	0	0	0	2	0	Bidar (2)
C16	2	B.6 (1)	1	1	0	0	0	1	1	Belagavi (2)
C17	2	B.6 (1)	0	1	1	0	0	1	1	Bagalkot (2)

*Lineages are shown with number of sequences assigned that lineage in brackets
**Clinical State – Asym: Asymptomatic at sample collection, Sym: Symptomatic
