# Supplementary material for: Genomic epidemiology reveals multiple introductions and spread of SARS-CoV-2 in the Indian state of Karnataka
Source: PLoS One. 2020 Dec 17;15(12):e0243412. doi: 10.1371/journal.pone.0243412 (PMC7746284; doi:10.1371/journal.pone.0243412)
Supplement: S6 Table — (PDF) [file pone.0243412.s008.pdf]

S6 Table. Lineage Reclassification.

Sr. No.	Pangolin Lineage*	Lineage Supported by ML tree**	Comments
1	B.26	B	Collapsed to parent
2	B.23	B.6	Reassigned
3	B.1.144	B.1	Collapsed to parent
4	B.1.148	B.1	Collapsed to parent
5	B.1.26	B.1	Collapsed to parent
6	B.23	B	Collapsed to parent
7	B.1.5	B.1	Collapsed to parent
8	B.1.36.1	B.1	Collapsed to parent
9	B.1.151	B.6	Reassigned
10	A	B.6	Reassigned
11	B.1.1.32	B.1.1	Collapsed to parent
12	B.1	B.1.80	Reassigned
13	B.1.150	B.1.80	Reassigned

*Pangolin v 2.07 lineages version 2020-08-29
**Maximum Likelihood Tree (Fig 2B)
