# Supplementary material for: Genomic epidemiology reveals multiple introductions and spread of SARS-CoV-2 in the Indian state of Karnataka
Source: PLoS One. 2020 Dec 17;15(12):e0243412. doi: 10.1371/journal.pone.0243412 (PMC7746284; doi:10.1371/journal.pone.0243412)
Supplement: S8 Table — (PDF) [file pone.0243412.s010.pdf]

S8 Table. Accession Numbers			
BioProject Accession ID. PRJNA670824			
Sr. No	GISAID Virus name	GISAID Accession ID	GenBank Accession ID
1	hCoV-19/India/KA-nimh-0113/2020	EPI_ISL_428479	MT396247
2	hCoV-19/India/KA-nimh-0116/2020	EPI_ISL_428480/515932	MW191497
3	hCoV-19/India/KA-nimh-0130/2020	EPI_ISL_428481	MT396243
4	hCoV-19/India/KA-nimh-0182/2020	EPI_ISL_428482	MT396242
5	hCoV-19/India/KA-nimh-0318/2020	EPI_ISL_428483	MT396246
6	hCoV-19/India/KA-nimh-0351/2020	EPI_ISL_428484	MT396245
7	hCoV-19/India/KA-nimh-0834/2020	EPI_ISL_428485	MW191498
8	hCoV-19/India/KA-nimh-0916/2020	EPI_ISL_515933	MW181790
9	hCoV-19/India/KA-nimh-0996/2020	EPI_ISL_428486	MT396248
10	hCoV-19/India/KA-nimh-0999/2020	EPI_ISL_486382	MW191499
11	hCoV-19/India/KA-nimh-1071/2020	EPI_ISL_428487	MT396244
12	hCoV-19/India/KA-nimh-1090/2020	EPI_ISL_516075	MW181791
13	hCoV-19/India/KA-nimh-1114/2020	EPI_ISL_516076	MW181792
14	hCoV-19/India/KA-nimh-1398/2020	EPI_ISL_515934	MW181793
15	hCoV-19/India/KA-nimh-1400/2020	EPI_ISL_515935	MW181794
16	hCoV-19/India/KA-nimh-1407/2020	EPI_ISL_515936	MW181795
17	hCoV-19/India/KA-nimh-1441/2020	EPI_ISL_591540	MW191500
18	hCoV-19/India/KA-nimh-1453/2020	EPI_ISL_516077	MW181796
19	hCoV-19/India/KA-nimh-1454/2020	EPI_ISL_486392	MW181797
20	hCoV-19/India/KA-nimh-1456/2020	EPI_ISL_583915	MW181798
21	hCoV-19/India/KA-nimh-1519/2020	EPI_ISL_515937	MW181799
22	hCoV-19/India/KA-nimh-1553/2020	EPI_ISL_486393	MW181800
23	hCoV-19/India/KA-nimh-1596/2020	EPI_ISL_436156	MT434759
24	hCoV-19/India/KA-nimh-1598/2020	EPI_ISL_436157	MT434760
25	hCoV-19/India/KA-nimh-1600/2020	EPI_ISL_516078	MW181801
26	hCoV-19/India/KA-nimh-2873/2020	EPI_ISL_436137	MT434757
27	hCoV-19/India/KA-nimh-3033/2020	EPI_ISL_515938	MW191501
28	hCoV-19/India/KA-nimh-3045/2020	EPI_ISL_515939	MW191502
29	hCoV-19/India/KA-nimh-3047/2020	EPI_ISL_515940	MW181802
30	hCoV-19/India/KA-nimh-3720/2020	EPI_ISL_486394	MW181802
31	hCoV-19/India/KA-nimh-3948/2020	EPI_ISL_515941	MW181803
32	hCoV-19/India/KA-nimh-3952/2020	EPI_ISL_436138	MT434758
33	hCoV-19/India/KA-nimh-3959/2020	EPI_ISL_486383	MW181805
34	hCoV-19/India/KA-nimh-3964/2020	EPI_ISL_515942	MW181806

35	hCoV-19/India/KA-nimh-3970/2020	EPI_ISL_436139	MT439597
36	hCoV-19/India/KA-nimh-3975/2020	EPI_ISL_486881	MW181807
37	hCoV-19/India/KA-nimh-4263/2020	EPI_ISL_486395	MW181808
38	hCoV-19/India/KA-nimh-4376/2020	EPI_ISL_436140	MT439595
39	hCoV-19/India/KA-nimh-4377/2020	EPI_ISL_486396	MW181809
40	hCoV-19/India/KA-nimh-4378/2020	EPI_ISL_436141	MT439596
41	hCoV-19/India/KA-nimh-5102/2020	EPI_ISL_486397	MW181810
42	hCoV-19/India/KA-nimh-5297/2020	EPI_ISL_486398	MW181811
43	hCoV-19/India/KA-nimh-7139/2020	EPI_ISL_515943	MW181812
44	hCoV-19/India/KA-nimh-7151/2020	EPI_ISL_515944	MW181813
45	hCoV-19/India/KA-nimh-7817/2020	EPI_ISL_486399	MW181814
46	hCoV-19/India/KA-nimh-8863/2020	EPI_ISL_486384	MW181815
47	hCoV-19/India/KA-nimh-10102/2020	EPI_ISL_486385	MW181816
48	hCoV-19/India/KA-nimh-10106/2020	EPI_ISL_486386	MW181817
49	hCoV-19/India/KA-nimh-10559/2020	EPI_ISL_486400	MW193967
50	hCoV-19/India/KA-nimh-11068/2020	EPI_ISL_515945	MW181818
51	hCoV-19/India/KA-nimh-11069/2020	EPI_ISL_515946	MW181819
52	hCoV-19/India/KA-nimh-11070/2020	EPI_ISL_486401	MW181820
53	hCoV-19/India/KA-nimh-11071/2020	EPI_ISL_515947	MW181821
54	hCoV-19/India/KA-nimh-11074/2020	EPI_ISL_515948	MW181822
55	hCoV-19/India/KA-nimh-11076/2020	EPI_ISL_486402	MW181823
56	hCoV-19/India/KA-nimh-11078/2020	EPI_ISL_583914	MW181824
57	hCoV-19/India/KA-nimh-11122/2020	EPI_ISL_486387	MW181825
58	hCoV-19/India/KA-nimh-11164/2020	EPI_ISL_486403	MW181826
59	hCoV-19/India/KA-nimh-11194/2020	EPI_ISL_515949	MW181827
60	hCoV-19/India/KA-nimh-11866/2020	EPI_ISL_515950	MW191503
61	hCoV-19/India/KA-nimh-11867/2020	EPI_ISL_515951	MW191504
62	hCoV-19/India/KA-nimh-11872/2020	EPI_ISL_515952	MW181828
63	hCoV-19/India/KA-nimh-11877/2020	EPI_ISL_486404	MW191505
64	hCoV-19/India/KA-nimh-12171/2020	EPI_ISL_515953	MW193966
65	hCoV-19/India/KA-nimh-13855/2020	EPI_ISL_515954	MW181829
66	hCoV-19/India/KA-nimh-13894/2020	EPI_ISL_515955	MW191506
67	hCoV-19/India/KA-nimh-14359/2020	EPI_ISL_515956	MW181830
68	hCoV-19/India/KA-nimh-14709/2020	EPI_ISL_486405	MW181831
69	hCoV-19/India/KA-nimh-14712/2020	EPI_ISL_486406	MW191507
70	hCoV-19/India/KA-nimh-14715/2020	EPI_ISL_486407	MW191508
71	hCoV-19/India/KA-nimh-14722/2020	EPI_ISL_515957	MW181832
72	hCoV-19/India/KA-nimh-14723/2020	EPI_ISL_486388	MW181833

73	hCoV-19/India/KA-nimh-14834/2020	EPI_ISL_515958	MW181834
74	hCoV-19/India/KA-nimh-14835/2020	EPI_ISL_515959	MW181835
75	hCoV-19/India/KA-nimh-15365/2020	EPI_ISL_515960	MW181836
76	hCoV-19/India/KA-nimh-15819/2020	EPI_ISL_486408	MW181837
77	hCoV-19/India/KA-nimh-15833/2020	EPI_ISL_515961	MW181838
78	hCoV-19/India/KA-nimh-15835/2020	EPI_ISL_486389	MW181839
79	hCoV-19/India/KA-nimh-15896/2020	EPI_ISL_486409	MW181840
80	hCoV-19/India/KA-nimh-15899/2020	EPI_ISL_515962	MW181841
81	hCoV-19/India/KA-nimh-17585/2020	EPI_ISL_515963	MW191509
82	hCoV-19/India/KA-nimh-17608/2020	EPI_ISL_515964	MW191510
83	hCoV-19/India/KA-nimh-17998/2020	EPI_ISL_515965	MW181842
84	hCoV-19/India/KA-nimh-19184/2020	EPI_ISL_515966	MW191511
85	hCoV-19/India/KA-nimh-19188/2020	EPI_ISL_515967	MW191512
86	hCoV-19/India/KA-nimh-19510/2020	EPI_ISL_515968	MW181843
87	hCoV-19/India/KA-nimh-19669/2020	EPI_ISL_515969	MW181844
88	hCoV-19/India/KA-nimh-19688/2020	EPI_ISL_515970	MW181845
89	hCoV-19/India/KA-nimh-19696/2020	EPI_ISL_515971	MW181846
90	hCoV-19/India/KA-nimh-19712/2020	EPI_ISL_515972	MW181847
91	hCoV-19/India/KA-nimh-19716/2020	EPI_ISL_515973	MW181848
