# Supplementary material for: Genomic epidemiology reveals multiple introductions and spread of SARS-CoV-2 in the Indian state of Karnataka
Source: PLoS One. 2020 Dec 17;15(12):e0243412. doi: 10.1371/journal.pone.0243412 (PMC7746284; doi:10.1371/journal.pone.0243412)

**S1 Appendix. Primers and Analysis Workflow.**

The Artic Network primer V3 were used for sequencing (https://github.com/artic-network/artic-ncov2019/tree/master/primer_schemes/nCoV-2019/V3). Gaps in the sequences were noted at four amplicon positions, additional primers were designed for these regions, a separate PCR reaction was set up to amplify these regions and sequencing was performed to fill these gaps. Details of primers are provided in the table below.

| Sl.no | Primer Name | Primer Sequence (5'- 3') | No of bases |
|---|---|---|---|
| 1 | V20_15K_L | CACCGTAGCTGGTGTCTCTA | 20 |
| | V20_15K_R | GCATCTCCTGATGAGGTTCCA | 21 |
| 2 | V20_5K_L | CAACATTAACCTCCACACGCA | 21 |
| | V20_5K_R | CACCAGCCCTTGCTCTGTAA | 20 |
| 3 | V20_26K_L | ACGGTTCATCCGGAGTTGTT | 20 |
| | V20_26K_R | GTACCGTTGGAATCTGCCAT | 20 |
| 4 | V20_28K_L | CTTTTGGTTCTCACTTGAACTGC | 23 |
| | V20_28K_R | CAACACGAACGTCATGATACTCT | 23 |

A schematic of the analysis workflow from sequencing reads to consensus generation is provided below.

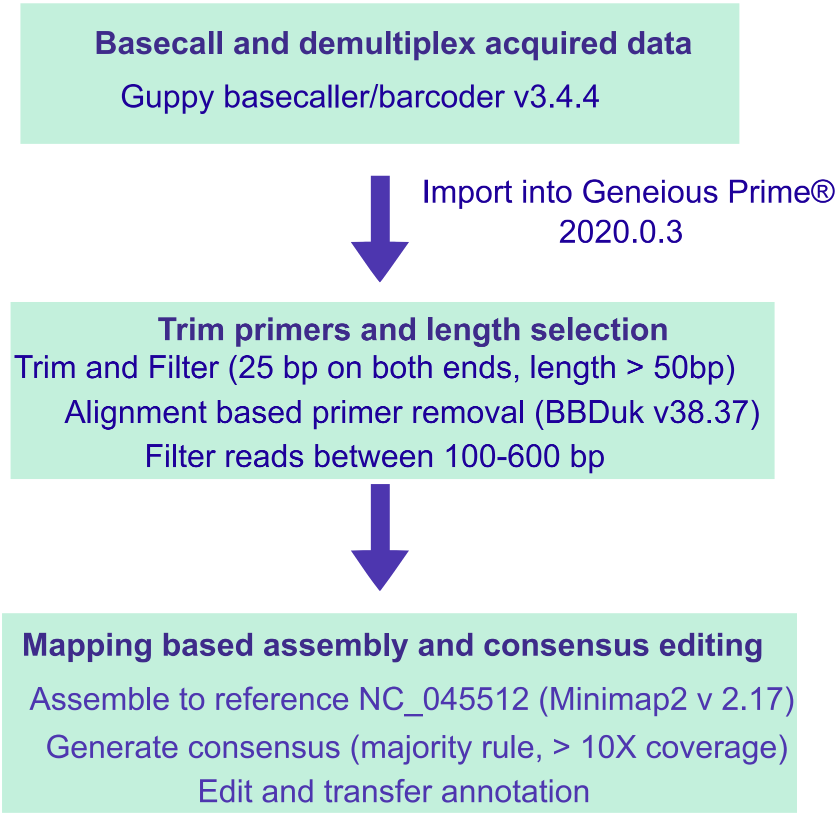

Supplement: S1 Appendix — (PDF) [file pone.0243412.s011.pdf]
